# A Novel *Shigella* Proteome Microarray Discriminates Targets of Human Antibody Reactivity following Oral Vaccination and Experimental Challenge

**DOI:** 10.1128/mSphere.00260-18

**Published:** 2018-08-01

**Authors:** Esther Ndungo, Arlo Randall, Tracy H. Hazen, Dane A. Kania, Krista Trappl-Kimmons, Xiaowu Liang, Eileen M. Barry, Karen L. Kotloff, Subhra Chakraborty, Sachin Mani, David A. Rasko, Marcela F. Pasetti

**Affiliations:** a Center for Vaccine Development and Global Health, University of Maryland School of Medicine, Baltimore, Maryland, USA; b Antigen Discovery, Inc., Irvine, California, USA; c Institute for Genome Sciences, University of Maryland School of Medicine, Baltimore, Maryland, USA; d Department of International Health, Johns Hopkins Bloomberg School of Public Health, Baltimore, Maryland, USA; e PATH, Washington, District of Columbia, USA; University of Kentucky

**Keywords:** *Shigella*, antibodies, proteome microarray, vaccines

## Abstract

*Shigella* spp. are a major cause of diarrhea and dysentery in children under 5 years old in the developing world. The development of an effective vaccine remains a public health priority, necessitating improved understanding of immune responses to *Shigella* and identification of protective antigens. We report the development of a core *Shigella* proteome microarray consisting of 2,133 antigen targets common to all *Shigella* species. We evaluated the microarray with serum samples from volunteers immunized with either an inactivated whole-cell S. flexneri serotype 2a (Sf2aWC) vaccine or a live attenuated S. flexneri 2a vaccine strain (CVD 1204) or challenged with wild-type S. flexneri 2a (Sf2a challenge). Baseline reactivities to most antigens were detected postintervention in all three groups. Similar immune profiles were observed after CVD 1204 vaccination and Sf2a challenge. Antigens with the largest increases in mean reactivity postintervention were members of the type three secretion system (T3SS), some of which are regarded as promising vaccine targets: these are the invasion plasmid antigens (Ipas) IpaB, IpaC, and IpaD. In addition, new immunogenic targets (IpaA, IpaH, and SepA) were identified. Importantly, immunoreactivities to antigens in the microarray correlated well with antibody titers determined by enzyme-linked immunosorbent assay (ELISA), validating the use of the microarray platform. Finally, our analysis uncovered an immune signature consisting of three conserved proteins (IpaA, IpaB, and IpaC) that was predictive of protection against shigellosis. In conclusion, the *Shigella* proteome microarray is a robust platform for interrogating serological reactivity to multiple antigens at once and identifying novel targets for the development of broadly protective vaccines.

**IMPORTANCE** Each year, more than 180 million cases of severe diarrhea caused by *Shigella* occur globally. Those affected (mostly children in poor regions) experience long-term sequelae that severely impair quality of life. Without a licensed vaccine, the burden of disease represents a daunting challenge. An improved understanding of immune responses to *Shigella* is necessary to support ongoing efforts to identify a safe and effective vaccine. We developed a microarray containing >2,000 proteins common to all *Shigella* species. Using sera from human adults who received a killed whole-cell or live attenuated vaccine or were experimentally challenged with virulent organisms, we identified new immune-reactive antigens and defined a T3SS protein signature associated with clinical protection.

## Abstract (precis)

Each year, more than 180 million cases of severe diarrhea caused by *Shigella* occur globally. Those affected (mostly children in poor regions) experience long-term sequelae that severely impair quality of life. Without a licensed vaccine, the burden of disease represents a daunting challenge. An improved understanding of immune responses to *Shigella* is necessary to support ongoing efforts to identify a safe and effective vaccine. We developed a microarray containing >2,000 proteins common to all *Shigella* species. Using sera from human adults who received a killed whole-cell or live attenuated vaccine or were experimentally challenged with virulent organisms, we identified new immune-reactive antigens and defined a T3SS protein signature associated with clinical protection.

## INTRODUCTION

Shigella spp. account for more than 180 million cases of diarrheal disease globally every year (1). Children living in poor areas of the world bear the greatest burden of disease (2, 3); *Shigella* ranks among the top three agents of moderate-to-severe diarrhea (MSD) and dysentery during the first 5 years of life and rises to the first etiology of MSD among toddlers. The majority of infections are caused by Shigella flexneri (15 serotypes) and Shigella sonnei (1 serotype), while Shigella dysenteriae serotype 1 (the other 13 serotypes rarely cause disease) is responsible for outbreaks and pandemics in crowded settings (4, 5). Isolates of the less common Shigella boydii (19 serotypes) have mostly been detected in the Indian subcontinent (2, 4). While the risk of infection can be reduced by facilitating access to clean water and adequate sanitation, identifying safe and effective prophylactic tools to prevent diarrhea and morbidity caused by *Shigella* remains a public health priority (2). No approved vaccine is currently available. Several promising candidates are in different phases of development, including live attenuated and killed whole-cell organisms, O-polysaccharide protein conjugates, and subunit vaccines, and some have advanced into human clinical trials with different levels of success (reviewed in references 4 and 6–9). An improved understanding of host immune responses to *Shigella* target antigens and immunological mechanisms required to prevent infection is necessary to inform vaccine development efforts.

While no definitive correlates of protection have been established, seroepidemiological studies have revealed strong associations between naturally acquired protective immunity or reduced risk of shigellosis and the levels of antibodies against the surface lipopolysaccharide (LPS) and the invasion plasmid antigens (Ipas) (10–16). Evidence from clinical and field trials and experiments in nonhuman primates indicates that *Shigella* (mainly O-polysaccharide)-induced immunity is serotype specific (17–19). Hence, a drawback of vaccine concepts that rely solely on LPS-induced immunity is the restricted coverage, thus requiring O-antigen combinations, which complicates clinical evaluation and manufacturing and increases costs. The pursuit of a broad-spectrum vaccine that can prevent disease caused by multiple serotypes compels the identification of target antigens common to widely circulating *Shigella* species/strains.

The goal of this study was to evaluate genomes of epidemiologically relevant *Shigella* isolates to identify protein targets of natural and vaccine-induced human immune responses. To this end, we developed a *Shigella* protein array based on an established high-throughput immune profiling platform, with an emphasis on conserved proteins, to identify immunogenic and reactive antigens that would be relevant for the development of vaccines and diagnostics. Microarrays have been successfully used to assay immune responses elicited by natural exposure to multiple pathogens (20, 21) or in response to vaccination (22) to improve vaccine development strategies, and in some cases, to predict immune signatures for protection (23, 24). The protein microarray allowed us to probe over 2,000 *Shigella* antigens in a single assay and characterize immune responses in volunteers following three different interventions: (i) vaccination with an inactivated whole-cell vaccine, (ii) vaccination with a live attenuated vaccine strain, and (iii) challenge with a clinically relevant virulent S. flexneri strain. This is the first description of a *Shigella* proteome array and systematic probing of *Shigella* core antigens for immune reactivities following vaccination and experimental infection in association with disease outcome.

## RESULTS

### Selection of *Shigella* core proteins for the microarray and development of the microarray.

We aimed to develop a microarray featuring *Shigella* core proteins common to all *Shigella* isolates, especially those circulating worldwide. To ensure broad representation of clinically relevant species, we performed a comparative bioinformatics analysis of over 500 sequenced and annotated *Shigella* genomes to identify targets with transmembrane regions, signal peptides, and lipoprotein motifs. To identify the common core of *Shigella*, we counterselected against diverse phylogenomic and pathovar representatives of the closely related Escherichia coli. A total of 1,857 genomic features were identified that represented the chromosomal core of *Shigella*. This number is similar to estimates of the conserved core of E. coli and *Shigella* isolates using other datasets (25, 26). Gene identifiers and DNA and amino acid sequences are presented in Table S1 in the supplemental material. Additional features included on the array were the complete coding sequence content from the pCP301 virulence plasmid from S. flexneri (27) and plasmid A from S. sonnei 53G (28).

10.1128/mSphere.00260-18.1 TABLE S1  Description of all antigens on the *Shigella* core proteome microarray. Download TABLE S1, XLS file, 2.7 MB. Copyright © 2018 Ndungo et al. 2018 Ndungo et al. This content is distributed under the terms of the Creative Commons Attribution 4.0 International license.

### Selection of clinical samples for the microarray.

To probe the microarray, we selected serum samples from human adult volunteers who had participated in *Shigella* vaccine and experimental challenge studies (Table 1). Two different vaccine modalities were selected (killed whole-cell and live attenuated organisms) to distinguish intrinsic differences in host immune responses. Individuals with preexisting immunity who were exposed to virulent organisms in an experimental challenge and experienced different degrees of illness were included to assess targets of immunity associated with clinical protection and disease severity (mild to severe).

**TABLE 1:** Selection of samples used to probe the microarray

Study no.	Intervention	Treatment; sample types(s) and days collected	Cohort categories	No(s). of subjects	Reference
1	Sf2aWC vaccine	Formalin-inactivated S. flexneri 2a whole-cell vaccine (10^11^ vp/ml)^a^; serum and ALS samples collected on days −1, 7, 35, and 63	Day −1 (prevaccination) and days 7, 35, 63	5	29
2	CVD 1204 vaccine	Live attenuated S. flexneri 2a strain 2457T with a genomic deletion in guanine nucleotide biosynthesis (Δ*guaBA*); sera collected on days −1 and 28	10^7^, 10^8^, and 10^9^ CFU	4, 2, and 5	30
3	Sf2a challenge	S. flexneri 2a challenge (10^3^ CFU); sera collected on days −1 and 28	DI 0, DI 1, DI 2, and DI 3^b^	4, 3, 4, and 3	68

a vp, vaccine particles (formalin-inactivated bacterial cells).

b DI, disease index; DI 0, healthy; DI 1, mild disease; DI 2, moderate disease; DI 3; severe disease.

Antibodies were measured in serum and in culture supernatants from mucosally primed antibody-secreting cells present in the circulation 7 to 10 days after oral vaccination (antibody in lymphocyte supernatant [ALS]). Serum and ALS samples corresponded to inactivated whole-cell S. flexneri serotype 2a (Sf2aWC) vaccine recipients prior to and 1 week after each vaccination. Individuals who had received the highest dosage levels were selected as the most robust responding cohort (29). Serum samples from live attenuated S. flexneri 2a vaccine strain (CVD 1204) recipients corresponded to prevaccination and 28 days postvaccination and included samples from individuals who received increasing dosage levels (30). Serum samples from individuals challenged with wild-type S. flexneri 2a (Sf2a challenge) were obtained before and 28 days postinfection and included samples from individuals who remained healthy (disease index [DI] 0) or experienced mild disease (DI 1), moderate disease (DI 2), or severe disease (DI 3) (31).

### Immune profiles. (i) Microarray responses to known vaccine target antigens.

**FIG 1:**
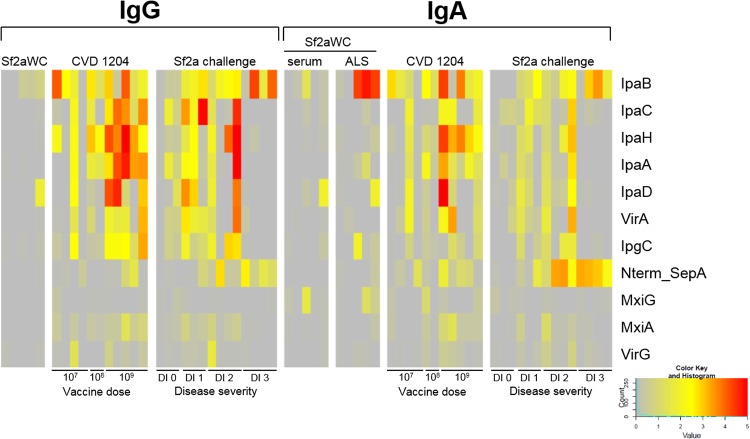
Heat map overview of IgG and IgA immune profiles in vaccinated and challenged individuals. Rows represent *Shigella* antigens probed, ranked by highest delta increases in signal intensity in individuals challenged with S. flexneri 2a (Sf2a challenge) from top to bottom. Columns represent individual serum or ALS samples; CVD 1204 samples are arranged by increasing dose from left to right, and Sf2a challenge samples are arranged by increasing disease severity (DI 0, healthy; DI 1, mild disease; DI 2, moderate disease; DI 3, severe disease) from left to right. The average difference in signal intensities is represented by the color shown in the key and reflects day 63 versus day −1 for Sf2aWC, day 28 versus day 0 for CVD 1204, and day 28 versus day 0 for Sf2a challenge.

To better interpret the responses shown by the microarray, we focused our analysis on antigens for which there were increased signal intensities following intervention, i.e., vaccination with Sf2aWC or CVD 1204, or Sf2a challenge. The signal intensities for samples in each group for each antigen were averaged, and the difference between the average intensities before and after vaccination or challenge was calculated to obtain the delta increase in signal reactivity. Figure 1 illustrates the resulting immune profile as a heat map of the top 10 antigens with the greatest delta increases ranked based on reactivity in response to Sf2a challenge. Delta increases of additional antigens (not represented in the heat map shown in Fig. 1) are provided in Tables S2 to S7.

10.1128/mSphere.00260-18.2 TABLE S2  Sf2aWC—normalized data. Download TABLE S2, XLS file, 2.7 MB. Copyright © 2018 Ndungo et al. 2018 Ndungo et al. This content is distributed under the terms of the Creative Commons Attribution 4.0 International license.

10.1128/mSphere.00260-18.3 TABLE S3  Sf2aWC—raw data. Download TABLE S3, XLS file, 1.5 MB. Copyright © 2018 Ndungo et al. 2018 Ndungo et al. This content is distributed under the terms of the Creative Commons Attribution 4.0 International license.

10.1128/mSphere.00260-18.4 TABLE S4  CVD 1204—normalized data. Download TABLE S4, XLS file, 2.1 MB. Copyright © 2018 Ndungo et al. 2018 Ndungo et al. This content is distributed under the terms of the Creative Commons Attribution 4.0 International license.

10.1128/mSphere.00260-18.5 TABLE S5  CVD 1204—raw data. Download TABLE S5, XLS file, 1 MB. Copyright © 2018 Ndungo et al. 2018 Ndungo et al. This content is distributed under the terms of the Creative Commons Attribution 4.0 International license.

10.1128/mSphere.00260-18.6 TABLE S6  S. flexneri 2a challenge—normalized data. Download TABLE S6, XLS file, 2.5 MB. Copyright © 2018 Ndungo et al. 2018 Ndungo et al. This content is distributed under the terms of the Creative Commons Attribution 4.0 International license.

10.1128/mSphere.00260-18.7 TABLE S7  S. flexneri 2a challenge—raw data. Download TABLE S7, XLS file, 1.2 MB. Copyright © 2018 Ndungo et al. 2018 Ndungo et al. This content is distributed under the terms of the Creative Commons Attribution 4.0 International license.

The overall reactivity was markedly higher in individuals orally exposed to the infecting organisms (Sf2a challenge) or live oral vaccine (CVD 1204) than in those exposed to the killed whole-cell vaccine (Sf2aWC). The top 10 antigens identified in the microarray represent members of the type three secretion system (T3SS), either as part of its architecture or as effectors. These include the invasion plasmid antigens (Ipas) IpaA, IpaB, IpaC, IpaD, and IpaH, the chaperone IpgC, and MxiA, MxiG, and VirG. Three of these top-10 proteins, IpaB, IpaC and IpaD, have been shown to be immunogenic in human recipients of live oral or Invaplex vaccines (30, 32, 33) and in experimentally infected individuals (31). Antibodies against these proteins, as well as to LPS, have been detected in sera from acute and convalescent patients (14, 16).

**FIG 2:**
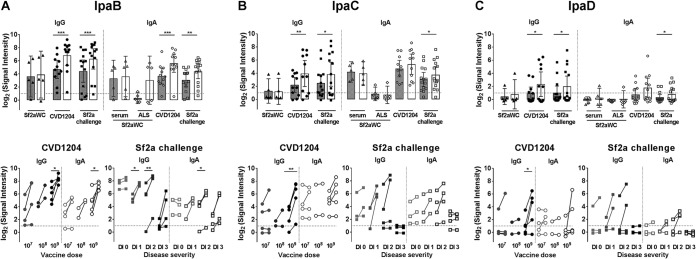
Immune profiles confirm IpaB, IpaC, and IpaD as immunogenic targets. Normalized signal intensities of IgG and IgA responses to IpaB (A), IpaC (B), and IpaD (C) in serum and ALS samples from individuals prior to (shaded bars) and following (open bars) vaccination with Sf2aWC or CVD 1204 and Sf2a challenge (top). Signal intensities were further dissected (bottom) based on increasing CVD 1204 vaccine dosage level or disease severity following Sf2a challenge. DI, disease index; DI 0, healthy; DI 1, mild disease; DI 2, moderate disease; DI 3, severe disease. Dotted line indicates threshold for reactivity, set as 1. Comparisons pre- versus postintervention were analyzed by paired *t* test (*, *P* < 0.05; **, *P* < 0.01; ***, *P* < 0.001).

When comparing responses to the IpaB, -C, and -D cluster, we found that overall, greater signal intensities were obtained for IpaB-specific IgG and IgA (Fig. 2A). This was clearly evidenced by the high serum reactivities in the CVD 1204-vaccinated and Sf2a-challenged individuals. The signal intensities in Sf2aWC-vaccinated subjects were lower overall and similar for IpaB, -C, and -D. Importantly, however, a substantial IpaB IgA signal increase was observed postvaccination in three of five ALS samples (Fig. 2A).

Among the CVD 1204 vaccine recipients, IpaB responses increased significantly for both IgG and IgA postvaccination in the highest-dosage (10^9^ CFU) group (Fig. 2A, bottom). The IpaB microarray responses were also analyzed in *Shigella*-challenged individuals based on disease outcomes. Statistically significant delta increases were seen in individuals who experienced mild or moderate disease (Fig. 2A, bottom). Individuals who remained healthy had high signal intensities before and after challenge and no seroconversion was observed, while for those with severe disease, two of four individuals had at least 4-fold increases in signal intensities postchallenge. The same serological trends had been observed for IpaB-specific antibodies measured by enzyme-linked immunosorbent assay (ELISA) and for serum bactericidal antibody (SBA) and opsonophagocytic killing assay (OPKA) titers in this group (31).

IgG responses to IpaC and IpaD were detected in the post-CVD 1204 vaccination (particularly in the high-dose group) and post-Sf2a challenge groups, although the signals were not as robust as those seen for IpaB; this trend was not as apparent with IgA responses (Fig. 2B and C). In general, IpaC signal responses were higher than those seen with IpaD.

### (ii) Microarray responses identifying new target antigens.

**FIG 3:**
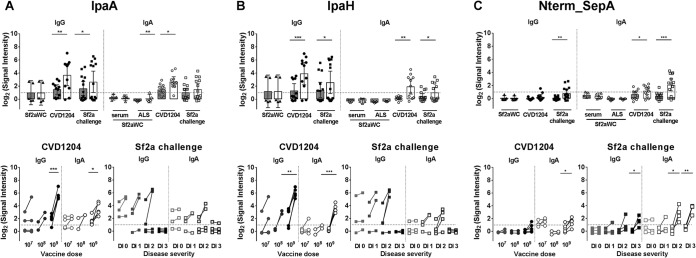
Identification of new immunogenic targets. Normalized signal intensities of IgG and IgA responses to newly discovered immunoreactive antigens IpaA (A), IpaH (B), and Nterm_SepA (C) in serum and ALS samples from individuals prior to (shaded bars) and following (open bars) vaccination with Sf2aWC or CVD 1204 and Sf2a challenge (top). Signal intensities were also examined based on increasing CVD 1204 vaccine dosage level or disease severity following Sf2a challenge (bottom) as described in the legend to Fig. 2. Dotted line indicates threshold for reactivity, set as 1. Comparisons pre- versus postintervention were analyzed by paired *t* test (*, *P* < 0.05; **, *P* < 0.01; ***, *P* < 0.001).

One of the main goals of the study was to identify novel antibody targets. To this end, we focused on the top 10 antigens that showed increased reactivity postintervention and that (to our knowledge) have not been considered among mainstream *Shigella* vaccine antigens. This was the case for IpaA, which participates in entry of effectors by the T3SS (34). Similar to the results for the other Ipa proteins, IpaA-specific IgG and IgA exhibited significant increases in reactivity post-CVD 1204 (but not Sf2aWC) vaccination (Fig. 3A). There was also a trend of increased IpaA-specific IgG and IgA signal intensities following Sf2a challenge (Fig. 3A, bottom). A noticeable difference from IpaB was the complete lack of responses in challenged individuals who experienced severe disease; this was also true for IpaC and IpaD.

A conserved IpaH ranked third among the top-10 antigens recognized by the CVD 1204 and Sf2a specimens (Fig. 3B). IpaH family proteins are present in all *Shigella* spp., and versions of this gene are used in PCR assays to identify *Shigella* infection in fecal samples (26, 35). The responses to the conserved IpaH followed the same pattern as those described above against IpaA; signal increases were detected post-CVD 1204 vaccination, particularly in the highest-dose group, and post-Sf2a challenge (although they did not reach statistical significance for any disease outcome and were blunted in the severe-disease group) (Fig. 3B, bottom).

Another notable antigen was the N-terminal region of *Shigella* extracellular protein A (SepA), hereinafter designated Nterm_SepA (Fig. 3C). SepA is a serine protease autotransporter (SPATE), similar to EatA from enterotoxigenic E. coli (ETEC) (36). Nterm_SepA reactivity was increased post-CVD 1204 (but not Sf2aWC) vaccination and post-Sf2a challenge (Fig. 3C). Notably, this was not observed for the C-terminal region or for the full-length SepA protein (data not shown). Nterm_SepA-specific IgA (but not IgG) signals were significantly increased in the highest-dose CVD 1204 recipients (Fig. 3C). Interestingly, there were significant increases in signal intensities for Sf2a-challenged individuals who developed moderate and severe disease, particularly for IgA (Fig. 3C, bottom), while no responses were seen in volunteers who remained healthy.

Though the other top 10 antigens (VirA, IpgC, MxiA, MxiG, and VirG) showed high delta increases in signal intensities, these did not translate to statistical significance postvaccination or postchallenge.

### Comparison between ELISA and microarray data.

**FIG 4:**
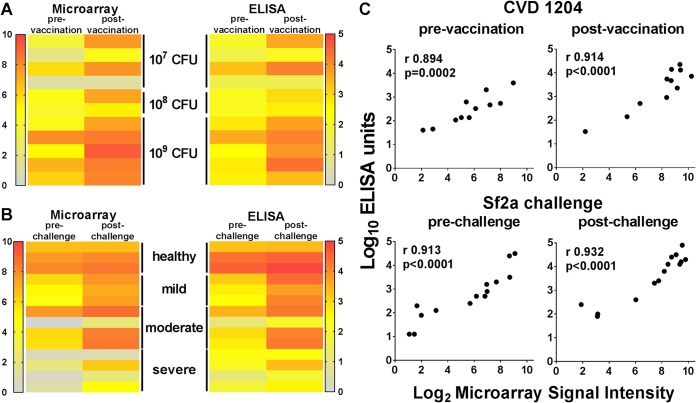
Normalized protein microarray signals are associated with ELISA titers. (A, B) Side-by-side comparison of normalized IpaB microarray signal intensities and mean IpaB ELISA units/ml measured in serum samples from individuals prior to and following CVD 1204 vaccination (A) and Sf2a challenge (B). (C) Association (Pearson’s correlation) between normalized IpaB protein microarray signals and IpaB-specific IgG ELISA titers prior to and following CVD 1204 vaccination and Sf2a challenge. Pearson’s correlation coefficients (*r*) and associated *P* values are indicated on the individual plots.

In contrast to traditional ELISAs, the proteins obtained by *in vitro* transcription and translation (IVTT) are not purified before printing on the microarray platform. Therefore, to validate the responses measured by the microarray, we juxtaposed normalized microarray signal reactivities for IpaB in CVD 1204 and Sf2a challenge samples to titers measured by ELISA (Fig. 4A and B). We found that both methods performed similarly in distinguishing serum reactivities in individuals with different disease outcomes post-Sf2a challenge (Fig. 4B) or those orally immunized with CVD 1204 (Fig. 4A). Strong correlations were also found between reactivity readouts obtained by both methods for IpaB in CVD 1204 and Sf2a challenge samples (Fig. 4C).

### A microarray signature as predictor of protective immunity.

**FIG 5:**
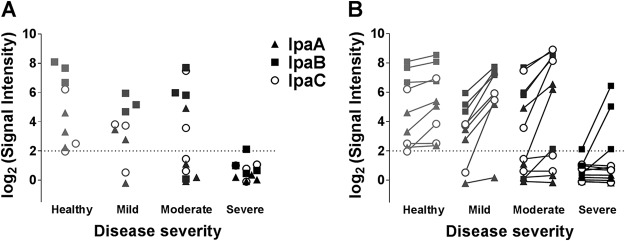
High reactivity to IpaA, IpaB, and IpaC in prechallenge samples is a predictor of protection from *Shigella* challenge. (A) Normalized signal intensities of IgG responses to invasion plasmid antigens (Ipas) IpaA, IpaB, and IpaC of individuals before Sf2a challenge, grouped according to disease outcomes postchallenge. Individuals who remained healthy post-*Shigella* challenge had high IgG signal intensities (>2) for response to invasion plasmid antigens IpaA, IpaB, and IpaC prechallenge. None of those who succumbed to severe disease had a signal intensity for response to IpaA, IpaB, or IpaC greater than 2 (marked by grid line). (B) Individual reactivities to IpaA, IpaB, and IpaC pre- and post-Sf2a challenge, grouped according to disease severity postchallenge.

Finally, having profiled antibody responses after vaccination and challenge, we asked whether these immune profiles could be used to identify patterns associated with clinical protection from severe disease. To do this, we considered antigens with the greatest intensities prechallenge in sera from individuals that remained healthy after Sf2a challenge. We found that individuals who remained healthy postinfection had high signal intensities (above a normalized signal intensity of 2) for the *Shigella* antigens IpaA, IpaB, and IpaC, while those who developed severe disease had lower signal intensities for these antigens prior to challenge (Fig. 5). The identification of this pattern, even with such a limited sample size, supports the relevance of this microarray platform and the possibility of defining serological signatures that could predict protective immunity against shigellosis.

## DISCUSSION

In this study, we report, for the first time, the development of a proteome microarray featuring antigens that make up the conserved core of the *Shigella* genome and demonstrated that such a tool can detect and distinguish a breadth of systemic and mucosally derived antibody responses to *Shigella* antigens. This microarray platform provides a foundation upon which to identify immunogenic responses to the *Shigella* core proteome.

Here, we characterized and compared the serological immune responses to a large number of *Shigella* antigens following three different interventions: an inactivated whole-cell S. flexneri 2a vaccine (Sf2aWC), a live attenuated S. flexneri 2a vaccine (CVD 1204), and experimental challenge with S. flexneri 2a (Sf2a challenge). The immune profile generated by CVD 1204 was similar to that of wild-type S. flexneri 2a challenge. This was reassuring, as this vaccine is derived by the deletion of the guanine nucleotide biosynthesis genes (Δ*guaBA*) from the parent challenge strain (2457T). Though significantly less virulent than 2457T, CVD 1204 was more reactogenic than expected (30). Further attenuation by deletion of *Shigella* enterotoxins 1 and 2 (ShET1 and ShET2) generated the subsequent candidate CVD 1208S, a leading vaccine candidate that was well tolerated while retaining high immunogenicity in humans (32, 37). The protective capacities of both the CVD 1204 and CVD 1208S vaccines remain to be determined.

Comparatively, a much lower response was seen in recipients of Sf2aWC, which could be explained by the fact that this is a killed vaccine. It is possible that the formalin used to inactivate the organism could have affected the integrity of the proteins in the vaccine particle, resulting in reduced responses to peptide antigens (38). In another study, immune responses to Ipa proteins were elicited by a formalin-inactivated S. sonnei whole-cell vaccine (SsWC), although this strain was specifically engineered to increase surface expression of protein antigens (39). Nonetheless, Sf2aWC has been shown to induce serum IgG responses to LPS, which peaked at day 7 (29), and was found to be protective in mouse and guinea pig models (38, 39). For this vaccine also, efficacy in humans is yet to be determined.

To identify key target antigens, we focused our analysis on increased mean signal intensity postintervention. The top-10 antigen list (representative of the greatest mean antibody responses) was populated with proteins that constitute the *Shigella* T3SS, a needlelike structure that promotes invasion of the host cell by delivering effector proteins from the bacterium into the host cytoplasm (40, 41). Of these, the microarray confirmed immunoreactivity to IpaB, -C, and -D, which have been included in several vaccine candidate approaches, including Invaplex (LPS plus IpaB, IpaC, and IpaD) (42); IpaB and IpaD delivered mucosally (43); IpaB and IpaD delivered parenterally with adjuvants (44–46); an IpaBD fusion (45); and IpaB, -C, and -D-containing outer membrane vesicles (47). Invaplex was shown to be immunogenic in humans (33), and the remaining candidates were shown to be protective in animals (reviewed in reference 8). Our results were also consistent with previous reports of statistically significant antibody-secreting-cell and serum antibody responses to all three antigens in CVD 1204 vaccine recipients (30). In addition, we have previously shown an association between elevated IpaB serum antibody levels and clinical protection in Sf2a-challenged volunteers (31). In aggregate, these findings support the validity of the microarray approach for identifying immunological targets relevant for *Shigella* infection and vaccination.

Two novel immunogenic proteins, IpaA and IpaH, which are also effectors secreted by the T3SS machinery, were identified in our analysis. IpaA is encoded within the same locus as IpaB to -D on the virulence plasmid and is likewise required for efficient invasion of *Shigella* by modulating host cell actin through its association with vinculin (34, 48, 49). The IpaH family of proteins are present in the genomes of the *Shigella* species, and for this reason, are routinely used as targets in real-time (RT)-PCR protocols to detect *Shigella* and/or the closely related enteroinvasive Escherichia coli (EIEC) in fecal samples (35, 50, 51). IpaA and IpaH have not been included in previous serological studies, and evidence of immunogenicity in humans has been limited. Western blot analyses have detected antibody responses to IpaA in *Shigella*-infected individuals, which were lower in magnitude than the responses to IpaB and IpaC (16, 52). In this study, we observed that serological reactivities to both antigens followed trends similar to that observed with IpaB, with increases post-CVD 1204 vaccination (i.e., all recipients of the highest dose had 4-fold seroconversions). In addition, as with IpaB, most of the volunteers who had either mild or moderate disease following Sf2a challenge had a 4-fold increase in IpaA and IpaH signal intensities, and reactive antibodies were already present prechallenge in individuals who remained healthy postinfection. These similarities suggest IpaA and IpaH might also be associated with protective immunity and are worth exploring as vaccine candidates.

The N-terminal region of SepA (Nterm_SepA) elicited striking (>4-fold) increases in IgA intensity in sera from volunteers who had moderate or severe disease following challenge. This was the only antigen, other than IpaB, for which we observed significant antibody increases in subjects who experienced severe disease. Unlike the Ipas, SepA is secreted independently of the T3SS and belongs to the serine protease autotransporters of *Enterobacteriaceae* (SPATEs) family of extracellular proteases produced by E. coli and *Shigella* spp. (reviewed in references 36 and 53). The N-terminal region, to which high-signal-intensity responses were observed, is the secreted portion of the protein that encodes the serine protease activity; the role of the protease in *Shigella* pathogenesis is ill defined, but it is likely important for virulence (36). While antibodies to other SPATEs have been detected in sera from patients (Pic and Pet [54] and SigA [55]), this is the first demonstration of antibodies reactive to SepA in human serum. Interestingly, SepA has more than 80% homology to EatA, a SPATE secreted by ETEC that has been shown to be immunogenic and protective against ETEC in a mouse model (56, 57).

A gap in knowledge for vaccine development and evaluation is our incomplete understanding of the immune mechanisms that prevent *Shigella* infection and the lack of firm immunological correlates of protection. We therefore interrogated our data set to define the specificity of serum antibodies at the time of challenge in relation to clinical disease postchallenge. Interestingly, we observed that, in aggregate, volunteers that remained healthy had elevated levels of T3SS effectors, specifically IpaA, IpaB, and IpaC, while those that had severe disease had almost undetectable levels of these three antigens. The protective baseline immunity observed in some of the enrolled individuals likely derives from natural exposure; participants were not serologically screened prior to enrollment, and the challenge group even included veterans who had likely been in contact with *Shigella* spp. Hence, the microarray could serve as a screening tool to discriminate naive (susceptible) versus immune individuals for purposes of enrollment in vaccine evaluation and challenge studies. We anticipate that further studies and larger sample sizes will be required to confirm our findings and validate these antigens as either true or surrogate correlates of protection. In a previous study, antibodies against VirG were associated with reduced disease postchallenge (31); VirG had positive signals in the microarray study but did not reach the top 10 among proteins with the highest increases in antibody reactivity postintervention.

Another contribution of our study was harmonizing the evaluation of immune responses across multiple studies, which has been advocated for as a better and more consistent approach for interpretation of data and advancement in the field (58). All clinical samples were processed and analyzed on the same platform, allowing simultaneous analysis of a broad range of antigens following multiple interventions (both vaccination and experimental infection) using one standardized methodology. Henceforth, the microarray can be useful for a thorough characterization of the antibody repertoire in individuals in relation to exposure, vaccination, and/or clinical protection. Importantly, the results obtained from the microarray platform correlated well with data obtained from conventional ELISA assays. This observation not only confirms the validity of the assay but also highlights the utility of the *in vitro* transcription and translation (IVTT) process to produce antigens that may be difficult to purify, essentially expanding the repertoire of *Shigella* antigens that can be examined.

One drawback of the *Shigella* core proteome microarray is that by focusing on antigens common to all *Shigella* species, protective antigens from some species or genomic clades may not be represented. Next steps include the production of species-specific arrays; by combining results from multiple arrays, a larger/refined pool of relevant antigens could emerge. The arrays used in this study did not include *Shigella* LPS, but future expanded versions could include species-specific O-polysaccharide variants. This would increase the utility of the microarray platform, since LPS is known to be a protective antigen and LPS-based vaccines candidates are advancing in clinical development, with recent studies confirming immunogenicity and efficacy in controlled human infection models (59, 60). The protein microarray can also help identify novel protective antigens for conjugation to LPS to improve LPS-based vaccine performance. Another limitation of our study is the relatively small sample sizes of the cohorts. Future studies with a larger sample size and an advanced microarray (including additional antigens) are planned. Notwithstanding, our results are relevant, as they confirmed immunogenic vaccine candidate antigens and revealed potential new ones.

In conclusion, we described the first immunoprofiling of the conserved core of the *Shigella* proteome and confirmed targets of *Shigella*-specific human immune responses that are possibly relevant for protection, as well as discovering additional such targets. The microarray is suitable for rapid and broad serologic screening of *Shigella* protein antigens in human clinical (or other *in vivo*) studies.

## MATERIALS AND METHODS

### Bioinformatic analysis of genomic data.

Genes were selected from sequenced isolates of *Shigella* species, with each of the four *Shigella* species represented, including 357 isolates of S. flexneri, 114 isolates of S. sonnei, 26 isolates of S. dysenteriae, and 44 isolates of S. boydii. The genome contents of the 541 *Shigella* species isolates were compared using the large-scale BLAST score ratio analysis (61), and encoded products that were common in all 451 isolates, as well as predicted to contain a signal for surface exposure, were identified. A total of 13,581 genomic features were identified in these genomes. A surface localization motif was identified using three prediction algorithms: PSORT (62), TMHMM (63, 64), and signalP (65, 66). To distinguish antigens specific to *Shigella* and lacking in the closely related Escherichia coli, the conserved core antigens were negatively selected against a collection of diverse E. coli pathovar isolates that represent each of the pathovars, as well as broad phylogenomic distribution. The antigens that were present in >70% of the *Shigella* species isolates and in <30% of the E. coli isolates were retained as the core *Shigella* genome. This gene set contains 1,857 conserved core genomic features of the *Shigella* chromosome, which were combined with 277 features from representative *Shigella* virulence plasmids of S. flexneri and S. sonnei. The plasmids of *Shigella* are often lost during culture and passage and, thus, are missing from the identified conserved core proteome but are believed to encode key virulence factors. As such, we included the complete set of coding regions from the S. flexneri 2a strain 301 plasmid pCP301 (GenBank accession number AF386526), as well as the S. sonnei 53G plasmid A (GenBank accession number NC_016833) on the microarray. The gene identifiers, as well as the sequences and isolates used as the templates for isolation, are included in Table S1 in the supplemental material. In addition to these informatically selected antigens, two purified proteins, Shiga toxin type 1 toxoid and Shiga toxin type 2 toxoid (BEI Resources, Manassas, VA), were included on the microarray. The strategy described considered a broad representation of the diversity of *Shigella* species and captured the proteome core in the microarray design.

### Protein microarray construction.

A clone library was constructed targeting all 2,134 complete genes, as well as 39 partial segments of these same genes, for a total of 2,174 cloning targets. Partial targets were added if the complete gene was over 3,000 bp, by splitting the gene into equal segments with an overlap of 500 bp. The partial genes are indicated by the suffix “_sX” added to the identifiers, where X is the index of the segment. Gene identifiers and DNA and amino acid sequences are presented in Table S1 in the supplemental material. Briefly, the clone library was created through an *in vivo* recombination cloning process with PCR-amplified coding sequences, and a complementary linearized expressed vector transformed into chemically competent E. coli cells was amplified by PCR and cloned into the pXI vector using a high-throughput PCR recombination cloning method. The cloning methodology is described in detail elsewhere (67). All 2,174 clones were sequenced (Retrogen, Inc., San Diego, CA), and the results matched the correct target for 2,133 clones; the antibody probing described in this study is limited to this set.

From each clone, the corresponding protein was expressed using an *in vitro* transcription and translation (IVTT) system, the E. coli cell-free rapid translation system (RTS) kit (5 Prime, Gaithersburg, MD), as previously described (67). Each expressed protein includes a 5′ polyhistidine epitope tag and a 3′ hemagglutinin (HA) epitope tag. After expressing the proteins according to the manufacturer’s instructions, translated proteins were printed onto nitrocellulose-coated glass AVID slides (Grace Bio-Labs, Inc., Bend, OR) using an OmniGrid accent robotic microarray printer (Digilabs, Inc., Marlborough, MA). Each slide contained three nitrocellulose pads on which the full array was printed (this allowed three samples to be probed per slide using sealed chambers that isolate the arrays). The printer head consists of 16 pins arranged in a 4-by-4 grid, which allowed for the printing of 16 array spots (primarily the expressed proteins, but also controls) concurrently, with one spot in each of 16 subarrays. Each set of 16 spots was printed on the three arrays (pads) of the first slide consecutively, then all three arrays on the second slide, and so on for an entire batch of slides. Microarray chip printing and protein expression were quality checked by probing random slides with anti-His and anti-HA monoclonal antibodies with fluorescent labeling.

### Clinical studies and samples used for study.

Serum samples to test the microarray were obtained from three previous clinical studies performed on healthy community volunteers at the Center for Immunization Research (Johns Hopkins University) or at the Center for Vaccine Development (University of Maryland, Baltimore) under approved IRB protocols. They are listed in Table 1 as follows. (i) Serum samples were collected from 5 subjects orally immunized with inactivated whole-cell S. flexneri serotype 2a vaccine (Sf2aWC) (29). Volunteers received 3 doses of 2.6 ± 0.8 × 10^11^ vaccine particles (vp)/ml, and serum was collected at day −1 (before vaccination) and 7 days after each dose (days 7, 35, and 63 postvaccination). Peripheral blood mononuclear cell (PBMC) culture supernatant was also obtained at the same time points for measurement of antibodies in lymphocyte supernatant (ALS). Both serum and ALS samples were used to probe the array. (ii) Serum samples were collected from 11 subjects orally immunized with a single dose of either 1 × 10^7^, 1 × 10^8^, or 1 × 10^9^ CFU of live attenuated S. flexneri 2a vaccine strain CVD 1204, which harbors deletion mutations in genes encoding enzymes in the guanine nucleotide synthesis pathway (Δ*guaBA*), in a phase I clinical study (30). Serum samples collected at days −1 (prior to vaccination) and day 28 (postvaccination) were used. (iii) Serum samples were obtained at days −1 (prior to challenge) and 28 (postchallenge) from 14 volunteer subjects who were fed 1 × 10^3^ CFU of the wild-type strain S. flexneri 2a strain 2457T as described previously (68); some of these volunteers had been previously vaccinated or were veterans, and they had various degrees of immunity. Specimens were selected from volunteers who remained healthy, as well as from those who experienced mild, moderate, and severe disease, as previously described (31). The number of samples tested was determined based on the microarray slides available.

### Proteome microarray probing.

Serum samples were diluted 1:100 and ALS samples were diluted 1:2 in a 3-mg/ml E. coli lysate solution (Antigen Discovery, Inc., Irvine, CA) in protein arraying buffer (Maine Manufacturing, Sanford, ME) and incubated at room temperature for 30 min. Arrays were rehydrated in blocking buffer for 30 min. The blocking buffer was removed, and arrays were probed with pretreated serum samples using sealed, fitted slide chambers to avoid cross-contamination between arrays. Arrays were incubated overnight at 4°C with agitation, washed five times with Tris-buffered saline (TBS)–0.05% Tween 20, and incubated with biotin-conjugated goat anti-human IgG (Jackson ImmunoResearch, West Grove, PA) diluted 1:200 in blocking buffer at room temperature. Arrays were washed three times with TBS–0.05% Tween 20 and incubated with streptavidin-conjugated SureLight P-3 (Columbia Biosciences, Frederick, MD) at room temperature, protected from light. Arrays were washed three times with TBS–0.05% Tween 20, three times with TBS, and once with water and then air dried by being centrifuged at 1,000 × *g* for 4 min and left overnight in a dessicator before scanning.

### Raw signal acquisition.

Probed microarrays (slides) were scanned using a GenePix 4300A high-resolution microarray scanner (Molecular Devices, Sunnyvale, CA), and an image file (.tiff) was saved for each array using GenePix pro 7 software. The signals in the scanned images were quantified using the Mapix software (Innopsys) autogridding feature. For this process, two input files are required: (i) a .gal file that defines the array and subarray layout, and (ii) the .tiff image file for an array. Once the autogridding is complete, the overlays of the mapped array, subarray, and individual spot locations are shown in the graphical user interface (GUI). If the automatic gridding fails to map to the correct positions, the mapping can be manually adjusted using the GUI. Once the gridding is confirmed to be correct, the array spots are quantified and saved to an output .gpr file. For each spot on the slide, the .gpr file contains the foreground intensity (median of pixels inside the circle defining the spot) and local background intensity (median of pixels just outside the circle defining the spot). The final raw intensity is the foreground intensity minus the local background intensity. The raw signals were automatically extracted and saved as .csv files in data matrix format, with array spots as rows and samples as columns, using R (http://www.R-project.org).

### Proteome microarray data normalization.

First, raw values were transformed using the base 2 logarithm. Next, the data set was normalized to remove systematic effects by subtracting the median signal intensity of the IVTT control spots for each sample. Since the IVTT control spots carry not only the chip, sample, and batch-level systematic effects, but also antibody background reactivity to the IVTT system, this procedure normalizes the data and provides a relative measure of the specific antibody binding versus the nonspecific antibody binding to the IVTT controls. With the normalized data, a value of 0.0 means that the intensity is no different than that of the IVTT controls, and a value of 1.0 indicates a doubling with respect to IVTT control spots.

### Purified protein ELISA antibody measurements.

Serum IgGs and IgAs specific for S. flexneri 2a invasion plasmid antigen B (IpaB) were measured by ELISA as previously described (30). Briefly, ELISA plates were coated with purified Ipa proteins at 0.1 µg/ml in phosphate-buffered saline (PBS), pH 7.2, for 3 h at 37°C, followed by blocking overnight at 4°C with 10% milk in PBS. Twofold dilutions of sera were tested in duplicate in 10% milk in PBS containing 0.05% Tween 20. Antigen-specific IgGs and IgAs were detected with horseradish peroxidase-labeled goat anti-human antibodies, followed by 3,3′,5,5′-tetramethylbenzidine (TMB) microwell peroxidase substrate (Kirkegaard & Perry Laboratories). Titers (ELISA units/ml) were reported as the reciprocal serum dilution that resulted in an absorbance value of 0.2 above the background value at 450 nm.
